# Complement C5a induces PD-L1 expression and acts in synergy with LPS through Erk1/2 and JNK signaling pathways

**DOI:** 10.1038/srep33346

**Published:** 2016-09-14

**Authors:** Ling-Ling An, Jacob V. Gorman, Geoffrey Stephens, Bonnie Swerdlow, Paul Warrener, Jessica Bonnell, Tomas Mustelin, Michael Fung, Roland Kolbeck

**Affiliations:** 1 Department of Respiratory, Inflammation and Autoimmunity, MedImmune, LLC. One Medimmune Way, Gaithersburg, MD 20878, USA; 2 Department of Infectious Diseases, MedImmune, LLC, Gaithersburg, MD 20878, USA

## Abstract

Severe bacterial infection results in both uncontrolled inflammation and immune suppression in septic patients. Although there is ample evidence that complement activation provokes overwhelming pro-inflammatory responses, whether or not it plays a role in immune suppression in this case is unclear. Here, we identify that complement C5a directly participates in negative regulation of immune responses to bacteria-induced inflammation in an *ex vivo* model of human whole blood. Challenge of whole blood with heat-killed *Pseudomonas aeruginosa* induces PD-L1 expression on monocytes and the production of IL-10 and TGF-β, which we show to be inhibited by C5a blockade. The induction of PD-L1 expression by C5a is *via* C5aR1but not C5aR2. Furthermore, C5a synergises with *P. aeruginosa* LPS in both PD-L1 expression and the production of IL-10 and TGF-β. Mechanistically, C5a contributes to the synergy in PD-L1 expression by specifically activating Erk1/2 and JNK signaling pathways. Our study reveals a new role for C5a in directly promoting immunosuppressive responses. Therefore, aberrant production of complement C5a during bacterial infection could have broader effect on compromising host defense including the induction of immune suppression.

Anaphylatoxin C5a generated from uncontrolled complement activation has been associated with inflammatory “cytokine storm” in sepsis patients^1^,^2^. C5a causes tissue damage by inducing pro-inflammatory cytokine and chemokine production, neutrophil chemoattraction and blood vessel leakage^3^,^4^. Recently, it was shown that C5aR1 knockout (C5aR1^−/−^) mice did not have lymphopenia following the induction of sepsis by cecal ligation and puncture (CLP), suggesting that C5a played an important role in regulation of adaptive immunity during the acute bacterial infection^5^.

The role of C5a in innate and adaptive immune regulation is complexed and heavily dependent on the local microenvironment. C5aR1 deficiency (C5aR1^−/−^) in dendritic cells (DCs) and CD4^+^ T cells led to the increase of TGF-β production and expansion of T regulatory cells (Tregs)^6^,^7^,^8^. On the other hand, C5a promoted IL-10 and TGF-β production from immature myeloid cells and favored the generation of Tregs^9^,^10^. C5a was also found to suppress inflammation by inhibition of IL-17A and IL-23 through the induction of IL-10 in an endotoxic shock model^11^.

One prominent characteristic of immune suppression in sepsis is the high level of program cell death 1 (PD-1) expressed on T cells^12^. Upon engaging with PD-1 ligand (PD-L1) on antigen presenting cells, the activated T cells become functionally impaired as an indication for poor prognosis of septic patients^13^,^14^. Since PD-L1 can be induced on primary monocytes^15^, which also express C5aR1 and C5aR2, we investigated whether C5a could be directly involved in PD-L1 dependent immune suppression during the acute inflammation. We employed an *ex vivo* human whole blood inflammation model in which complement activation was triggered when challenged with bacteria^16^. Here, we examined the direct effect of complement C5a on the modulation of PD-L1 expression and the production of IL-10 and TGF-β after human whole blood was challenged with heat-killed gram negative bacteria, *P. aeruginosa*. We confirmed the findings by direct stimulation of human primary monocytes with human plasma derived C5a. We further studied the interaction between C5a and *P. aeruginosa* LPS and identified their distinct signaling pathways in promoting PD-L1 expression.

## Results and Discussion

### C5a induces PD-L1 expression and the production of IL-10 and TGF-β

**Figure 1:**
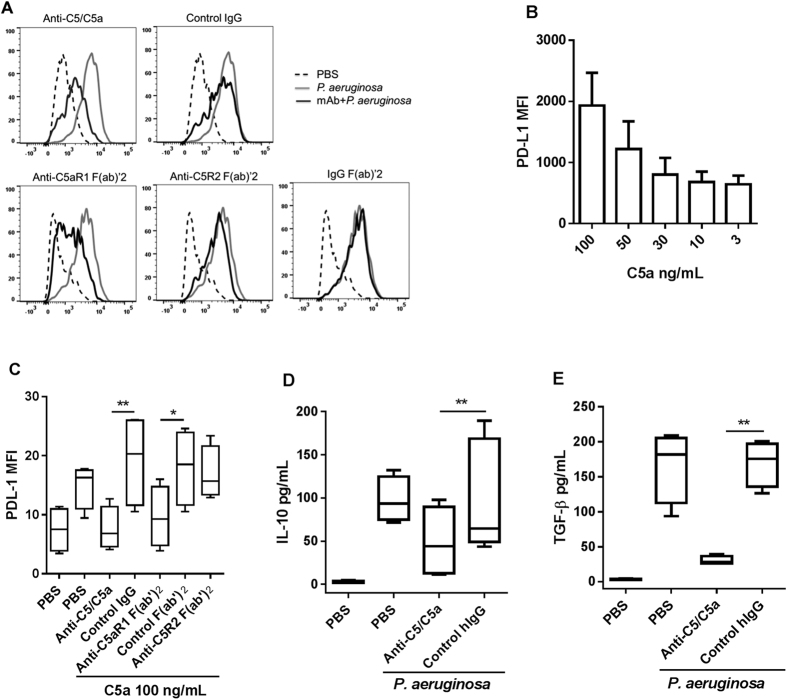
C5a induces PD-L1 expression on human monocytes. (**A**) Fresh human blood was incubated with IgG or F(ab’)_2_ at 130 nM for 30 min at 37 °C with 5% CO_2_ prior to the challenge with heat-inactivated *P. aeruginosa* at final concentration of 2 × 10^7^ cfu/mL; representative histogram of PD-L1 expression after 20 h incubation on gated CD14^+^ monocytes by flow cytometry (n = 10). (**B**) Freshly isolated human monocytes were treated with human plasma derived C5a for 20 h. (**C**) Primary human monocytes were pre-incubated with IgG or F(ab’)_2_ at 65 nM for 30 min before addition of C5a. n = 4 from different individual donors. (**D**,**E**) IL-10 or total TGF-β1 in the plasma was determined after 20 h incubation with *P. aeruginosa* in the presence of anti-C5/C5a or control antibody at 130 nM, n = 4-6 from different individual donors. Results represent the mean ± SEM. *p < 0.05, **p < 0.01 by one-way ANOVA followed by Sidak’s multiple comparisons test.

To determine the direct effect of complement C5a on immune suppression in *ex vivo*, we challenged freshly collected human whole blood with heat-killed *P. aeruginosa*, a highly relevant opportunistic pathogen in sepsis. By using dead bacteria, we avoided the impact on complement activation that could be caused by live bacteria-induced cytotoxicity and by LPS that directly released from live bacteria^16^. In this system, *P. aeruginosa* dose-dependently activated complement and generated C5a in the plasma after 10 min incubation at 37 °C (Supplementary Figure 1A). Clearly, PD-L1 expression was induced on monocytes after challenge with *P. aeruginosa* and the expression was inhibited in the presence of anti-C5/C5a IgG, a human monoclonal antibody specific to human C5 and C5a. The antibody blocks C5 and C5a binding to both C5aR1 and C5aR2^17^. Similarly, PD-L1 expression was inhibited by anti-C5aR1 F(ab’)_2_, but not anti-C5aR2 F(ab’)_2_ (Fig. 1A), indicating that *P. aeruginosa*-induced PD-L1 expression was dependent on C5a *via* C5aR1. To further confirm the finding, we incubated fresh human primary monocytes with human plasma derived C5a. Indeed, C5a alone dose-dependently induced PD-L1 expression (Fig. 1B). Similar to the finding in human whole blood, C5a-induced PD-L1 expression was significantly inhibited by blocking C5aR1 but not C5aR2 (Fig. 1C). Together, these data indicate that C5a generated from complement activation directly contributes to the induction of PD-L1 expression on monocytes.

In blood, C5a is quickly converted to C5adesArg by carboxypeptidases resulting in removing its C-terminal arginine (Arg) residue. Although it has about 100-fold lower binding affinity to C5aR1, C5adesArg is able to induce inflammation through C5aR1^18^. We therefore also examined the effect of C5adesArg on the regulation of PD-L1 expression on monocytes. Using natural human C5adesArg (Comp Tech), we found minimum effect in a side-by-side titration with C5a (Supplementary Figure 1B). This result suggests that C5a is the major player in regulation of PD-L1 expression. Considering that blocking C5aR2 showed no effect on PD-L1 expression on monocytes after challenge with *P. aeruginosa* in blood, the minimal activity shown by C5adesArg is unlikely related to its interaction with C5aR2. The difference found between C5a and C5adesArg in this case is not unexpected given that C5a and C5adesArg have been shown to have differential bioactivities upon binding to C5aR1. For example, C5a and C5adesArg have been shown to similarly induce IL-4 and IL-13 release by IL-3-cultured basophils however C5adesArg showed minimal effect on leukotriene C4 generation relative to C5a^19^. Additionally, C5a and C5adesArg are able to induce similar levels of IL-6 and TNFα expression by human monocyte-derived macrophages (HMDM), but C5a exposure of HMDM induces significantly more IL-10 production than C5adesArg stimulation^20^. The molecular mechanisms that account for the difference in C5a and C5adesArg activities are not yet clear. However, it has been shown that C5a and C5adesArg differentially interact with C5aR1^21^ and activate distinct signaling through G-proteins^18^.

Next, we determined the production of immune suppressive cytokines IL-10 and TGF-β. As shown in Fig. 1D,E, both IL-10 and total TGF-β1 levels in the plasma were elevated after 20 h incubation with *P. aeruginosa* and decreased in the presence of anti-C5/C5a antibody. To assess whether or not that IL-10 could directly regulate PD-L1 expression, we blocked IL-10 receptor with a neutralizing antibody before challenging with *P. aeruginosa*; we observed no significant inhibition of PD-L1 expression (data not shown), suggesting that PD-L1 and IL-10 have distinct functional pathways^22^,^23^. Noticeably, these immune suppressive responses occurred in the same time frame (20 h) as the inflammatory responses such as the production of IL-1β, IL-6, IL-8 and TNF-α (Supplementary Figure 1C), suggesting that C5a is concurrently responsible for both inflammatory and immune suppressive responses. In fact, innate immune suppression can occur during the acute phase of sepsis and contribute to the mortality in CLP-induced mouse sepsis model^24^.

In addition to monocytes, we also determined PD-L1 expression on other cell types including neutrophils, NK and T cells in the blood after challenge with heat-killed *P. aeruginosa*. PD-L1 expression was upregulated on neutrophils but not on other cell types. Challenge with heat-killed *P. aeruginosa* did not alter the expression of either CaR1 or CaR2 on monocyte, NKs or T cells. However, it decreased C5aR2 expression on neutrophils (Supplementary Figure 2). These data were summarized in Supplementary Table 1.

### C5a synergises with *P. aeruginosa* LPS in promoting PD-L1, IL-10 and TGF-β

**Figure 2:**
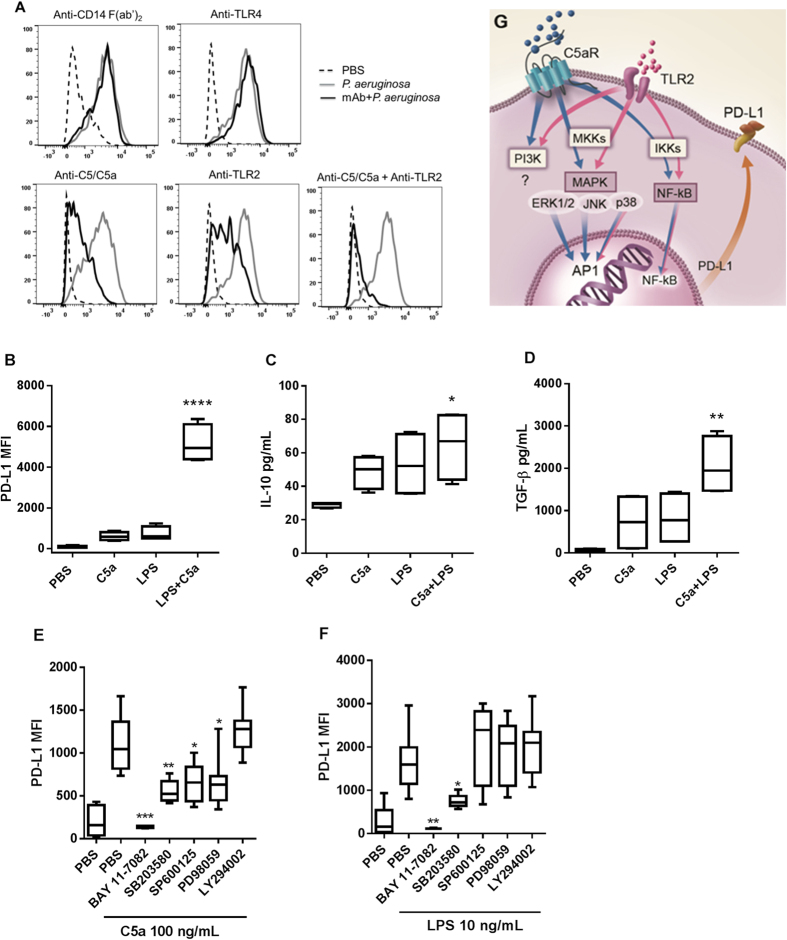
C5a synergises with *P. aeruginosa* LPS in PD-L1 expression though partially distinct signaling pathways. (**A**) Fresh human blood was incubated with IgG or F(ab’)_2_ at 130 nM for 30 min at 37 °C with 5% CO_2_ prior to the challenge with heat-inactivated *P. aeruginosa* at final concentration of 2 × 10^7^ cfu/mL. For combination treatment with anti-C5/C5a and anti-TLR2, 65 nM of each antibody was added; representative histogram of PD-L1 expression by flow cytometry (n = 4). (**B**–**D**) Freshly purified human monocytes were incubated with C5a (10 ng/mL) or LPS (1 ng/mL) or in combination for 20 h, PD-L1 expression on monocytes and IL-10 or total TGF-β1 in culture supernatant were determined (n = 4 from different individual donors, compared with C5a or LPS alone). (**E**,**F**) Monocytes were treated with BAY 11-7082 (10 μM), SB203589 (10 μM), SP600125 (10 μM), PD98059 (20 μM) or LY294002 (10 μM) for 30 min before adding C5a or LPS (n = 4–6, compared with C5a or LPS alone). Results represent the mean ± SEM. *p < 0.05, **p < 0.01, ***p < 0.005 and ****p < 0.001 by one-way ANOVA followed by Sidak’s multiple comparisons test. (**G**) Proposed mechanism of action of C5a and LPS in promoting PD-L1 expression on human monocytes.

Since *P. aeruginosa* LPS was also able to dose dependently induce PD-L1 expression (Supplementary Figure 3A), we then examined the functional relationship between complement C5a and LPS. Blocking CD14 or TLR4 showed no effect on *P. aeruginosa*-induced PD-L1 expression on monocytes in human blood. In contrast, blocking TLR2 significantly inhibited PD-L1 expression (Fig. 2A). The fact that the inhibition was induced *via* TLR2 was consistent with the findings from previous studies showing that *P. aeruginosa* mediated immune responses through TLR2 rather than TLR4^25^,^26^. Importantly, when both C5a and TLR2 were blocked, PD-L1 expression was reduced to the background level, indicating that both complement and TLR2 activation contribute to the PD-L1 expression in *P. aeruginosa*-induced inflammation. To further examine the interaction between C5a and LPS, we treated purified human monocytes at lower concentrations either with C5a or *P. aeruginosa* LPS alone or in combination. As shown, combination of C5a and LPS synergistically increased PD-L1 expression, IL-10 and TGF-β production (Fig. 2B–D), implying that C5aR1 and TLR2 likely acted through distinct but interactive pathways. These data are in agreement with early findings that C5aR1 and TLR2 or TLR4 synergized in inducing immune suppression, where cAMP was generated from macrophages in a *P. gingivalis* infection model^27^ and production of IL-6 and TNF-α was inhibited in human monocyte-derived macrophages^28^.

To dissect the contribution of various signaling pathways to C5a- and LPS-induced surface expression of PD-L1 on monocytes, we examined the role of each signaling pathway in PD-L1 expression by using pharmacological inhibitors. Previously, both C5a and LPS were shown to activate NF-κB, p38 MAPK, Erk1/2, JNK and PI3K signaling pathways in human monocytes^29^,^30^. Our data also showed that C5a and LPS at 100 ng/mL activated NF-κB, p38 MAPK, Erk1/2 and Akt signaling pathway in a time course study (Supplementary Figure 3B). All the inhibitors were used at the concentrations that showed no toxicity to the cells in a previous study^17^. As shown in Fig. 2E,F, both C5a and LPS induced PD-L1 expression through p38 MAPK and NF-κB signaling pathways as they were inhibited by SB203589 and BAY 11-7082, respectively. Blocking PI3 kinase with LY294002 did not show any effect on either C5a- or LPS-induced PD-L1 expression, indicating that PD-L1 upregulation is independent on PI3k/Akt pathway. Interestingly, when JNK or Erk1/2 signaling pathway was blocked by SP600125 or PD98059, respectively, only C5a- but not LPS-induced PD-L1 expression was significantly inhibited, indicating that C5a also induced PD-L1expression through activating these distinct signaling pathways. Therefore, the synergistic effect on PD-L1 expression by C5a and LPS is predominantly resulted from the distinct signaling pathways, although enhanced or additive p38 MAPK and NF-κB signaling by both could also play a role^31^. In addition to the fact that both C5aR1 and TLR2 were activated simultaneously through separate and/or enhanced signaling pathways, synergy can also be realized by cross-regulation between C5a and LPS through priming^17^,^28^,^32^. In fact, incubation of CD14^+^ human monocytes with C5a for 20 h increased TLR2 expression on cell surface (data not shown). Collectively, C5a and LPS synergistically promote PD-L1 expression on monocytes through both common signaling pathways (p38 MAPK and NF-κB) and C5a specific signaling pathways (Erk1/2 and JNK) as illustrated in Fig. 2G, with the condition that both C5aR1 and TLR2 are activated simultaneously.

Data have emerged that C5a exerts immune suppression through promoting regulatory mediators such as IL-10 and TGF-β from monocytes, macrophages and neutrophils^9^,^10^,^28^ and cAMP from macrophages^27^. Furthermore, the expression of immune suppression genes such as *Arg1*, *Ctla4*, *Il6*, *Il10*, *Lag3* and *Pdl1* were reduced in tumor tissue when C5aR1 was blocked^33^. Here we provided direct evidence that C5a alone was able to dose-dependently induce PD-L1 expression on human monocytes. Blocking C5a together with TLR2 completely abolished PD-L1 expression on monocytes in fresh human blood challenged with heat-killed *P. aeruginosa*. Although both C5a and LPS activate p38 MAPK and NF-κB signaling pathways, C5a alone specifically activates Erk1/2 and JNK signaling pathways to induce PD-L1 expression. Therefore, C5a and LPS synergise in PD-L1 expression mostly through independent signaling pathways. Furthermore, C5a and LPS synergistically induce the production of IL-10 and TGF-β from both human whole blood and purified monocytes. Together, these data suggest that in addition to pro-inflammatory responses, C5a plays an important role in generating immunosuppressive responses during the acute phase of gram negative bacteria-induced inflammation.

## Methods

### Reagents

The reagents used in this study were purchased or prepared as follows: heat-inactivated *P. aeruginosa* (AP01, InvivoGen); anti-human C5/C5a mAb (MedImmune)^17^; anti-human C5aR1 mAb (clone S5/1)^34^, anti-C5aR2 mAb (clone 1D9-M12)^35^, anti-TLR2 (clone T2.5), anti-TLR4 mAb (HTA125) and isotype control (BioLegend); F(ab’)_2_ were prepared by pepsin digestion (Pierce); plasma derived human C5a (Comp Tech); *P. aeruginosa* LPS and pharmacological inhibitors SB203589 (p38), BAY 11-7082 (NF-κB), LY294002 (PI3K), SP600125 (JNK) and PD98059 (Erk1/2) (Sigma Aldrich).

### Human blood and monocytes

The procedure for obtaining human blood from healthy volunteers was approved by Chesapeake Institutional Review Board. The informed consent was obtained from all volunteers. The All experiments were performed in accordance with the guidelines of MedImmune Review Committee for Clinical Research Studies. Donors with conditions that may affect basal level of PD-L1 expression were excluded from this study. Fresh human blood was collected using anti-coagulant lepirudin (Bayer) at 50 μg/mL^16^. Human primary monocytes were obtained from freshly isolated PBMCs using high recovery negative selection kit (STEMCELL Technologies).

### Stimulation of blood and monocytes

For human whole blood: to prevent complement activation, collected blood was quickly transferred to a sterile polypropylene reservoir (Brand Tech) and 80 μL was added to each well of a polypropylene 96-well U-bottom tissue culture plate (Costar). Anti-C5/C5a IgG, anti-C5aR F(ab’)_2_ or control (10 μL) was added to each well in triplicates. After incubation for 30 min at 37 °C with 5% CO_2_, heat-inactivated *P. aeruginosa* was added at final concentration of 2 × 10^7^ cfu/ml (10 μL). PD-L1 expression on CD14^+^ monocytes was detected after 20 h incubation.

For primary human monocytes: 3.0 × 10^5^ cells in 80 μL X-VIVO™ 15 serum-free medium (Lonza) were plated in each well of a U-bottom 96-well tissue culture plate. Cells pre-incubated with anti-C5/C5a IgG, anti-C5aR F(ab’)_2_ or inhibitor (10 μL) for 30 min at 37 °C with 5% CO_2_ before addition of C5a or LPS (10 μL) at final concentration of 100 ng/mL or 10 ng/mL. The expression of PD-L1 was detected after incubation for 20 h.

### Detection of PD-L1 expression

For human whole blood, cells were stained with APC conjugated anti-PD-L1 (clone 29E.2A3) and FITC conjugated anti-human CD14 (clone HCD14) (BioLegend) as recommended by the manufacturer and incubated for 20 min at room temperature. Red blood cells were then lysed using BD FACS Lysing Solution (BD Biosciences) and washed. For purified monocytes, Human TruStain FcX™ (Biolegend) was added to each well (5 μL/well) and incubated for 10 min at room temperature before staining. Live cells were gated for DAPI negative and CD14^+^; PD-L1 median fluorescence intensity (MFI) was determined by flow cytometry using Flowjo software (TreeStar).

### Detection of cytokines

Plasma or supernatant from overnight culture were collected. IL-10 level was determined using Quantikine ELISA kit (R&D). Total TGF-β1 was determined using DuoSet kit (R&D).

### Statistics

Data were analyzed by one-way ANOVA followed by Sidak’s test. *p* values adjusted for multiple testing are reported. A *p*-value less than 0.05 are considered significant (Prism, Graphpad).

## Additional Information

**How to cite this article**: An, L.-L. *et al*. Complement C5a induces PD-L1 expression and acts in synergy with LPS through Erk1/2 and JNK signaling pathways. *Sci. Rep*. **6**, 33346; doi: 10.1038/srep33346 (2016).

## Supplementary Material

Supplementary Information
