# Supplementary material for: Complement C5a induces PD-L1 expression and acts in synergy with LPS through Erk1/2 and JNK signaling pathways
Source: Sci Rep. 2016 Sep 14;6:33346. doi: 10.1038/srep33346 (PMC5022031; doi:10.1038/srep33346)

Complement C5a induces PD-L1 expression and acts in synergy with LPS through Erk1/2 and JNK signaling pathways

Ling-Ling An[1]*, Jacob V. Gorman[1], Geoffrey Stephens[1], Bonnie Swerdlow[1], Paul Warrener[2], Jessica Bonnell[2], Tomas Mustelin[1], Michael Fung[1] and Roland Kolbeck[1]

[1]Department of Respiratory, Inflammation and Autoimmunity; [2]Department of Infectious Diseases, MedImmune, LLC, Gaithersburg, MD 20878, USA

Supplementary Information

Methods

Reagents

Human plasma C5a and C5adesArg were from Comp Tech. PE-Cy7-anti-human C5aR1 (S5/1) and PE-anti-human C5aR2 (1D9-M12) were from BioLegend. Phospho-specific antibodies, Total IκBα (25/IκBα/MAD-3), p38 MAPK (pT183/pY182) (36/p38), p44/42 ERK1/2 (pT202/pY204) (20A) and Akt (M89-61), were from BD Biosciences.

Competent activation in human blood

Fresh human blood (80 uL) was added to each well of a 96-well polypropylene plate. For induction of C5a, serially diluted heat-killed *P. aeruginosa* in PBS (20 µL) was added to each well. The plate was incubated by floating on a 37°C water bath for 10 min. After centrifugation at 4°C for 10 min at 2000 rpm, plasma was collected in a 96-well polypropylene plate containing FUT-175 to prevent further complement activation (final concentration at 50 µg/mL, BD Biosciences). Plasma C5a was measured using Hu C5a OptEIA ELISA Kit II (BD Biosciences). For induction of cytokines and chemokines, anti-C5/C5a IgG or control IgG was added to each well in triplicates at 130 nM (final concentration). After incubation for 30 min at 37°C with 5% CO_2, heat-killed *P. aeruginosa* (10 µL) was added to each well at final concentration at $2x10^7$ cfu/mL. Plasma was collected after 20 h incubation at 37°C with 5% CO_2; cytokines and chemokines were determined using multiplex kit (Meso Scale Discovery).

Activation of signaling pathways

Human monocytes were plated 3-5x10^5 per well in 80 µL X-VIVO™ 15 serum-free medium in a U-bottom 96 well tissue culture plate and sit for 2 h at 37°C with 5% CO_2. LPS or C5a (final concentration at 100 ng/mL) was added to each well and incubated for (0, 2, 5 and 15 min) at 37°C with 5% CO_2. Activation was stopped by adding equal volume of Cytofix Buffer (BD Biosciences) for 10 min at the 37°C. After fixation cells were frozen at -80°C overnight. The following day monocytes were thawed at 37°C and permeabilized with -20°C Perm Buffer III (BD Biosciences) on ice for 30 min. After permeabilization cells were washed with 3% FBS in PBS and stained with phospho-specific antibodies for 30 minutes at room temperature prior flow cytometry analysis.

Supplementary Figure Legends

Supplementary Figure 1. (A) Quantification of plasma C5a induced by *P. aeruginosa* 10 min after incaution at 37°C in freshly collected human blood. The graph is representative of more than three independent experiments with different healthy donors. Bars represent mean ± SEM from triplicate wells. (B) PD-L1 expression on primary monocytes after incubation with C5a or C5adesArg for 20 h at 37°C with 5% CO_2 by flow cytometry (n=3). (C) Quantification of cytokines and chemokine induced by *P. aeruginosa* in the presence of anti-C5/C5a or control IgG after 20 h incubation, n=4-6 from different individual donors. Results represent the mean ± SEM. **p* <0.05, ***p*<0.01 and ****p*<0.005 by one-way ANOVA followed by Sidak's multiple comparisons test.

Supplementary Figure 2. The expression of PD-L1 (top), C5aR1(middle) and C5aR2 (bottom) after fresh human blood was challenged with heat-killed *P. aeruginosa* and incubated for 20 h at 37°C with 5% CO_2 by flow cytometry. Data were from 3 healthy donors.

Table1. Summary of FACS data from supplementary Figure 2.

Supplementary Figure 3. (A) Quantification of the expression of PD-L1 on human primary monocytes induced by *P. aeruginosa* LPS by flow cytometry after 20 h incubation. The graph is a representative of two independent experiments with different healthy donors. Bars represent mean ± SEM from triplicate wells. (B) Time course study of signaling pathway activation by C5a or LPS at100 ng/mL by flow cytometry (n=3).

Supplementary Figure 1

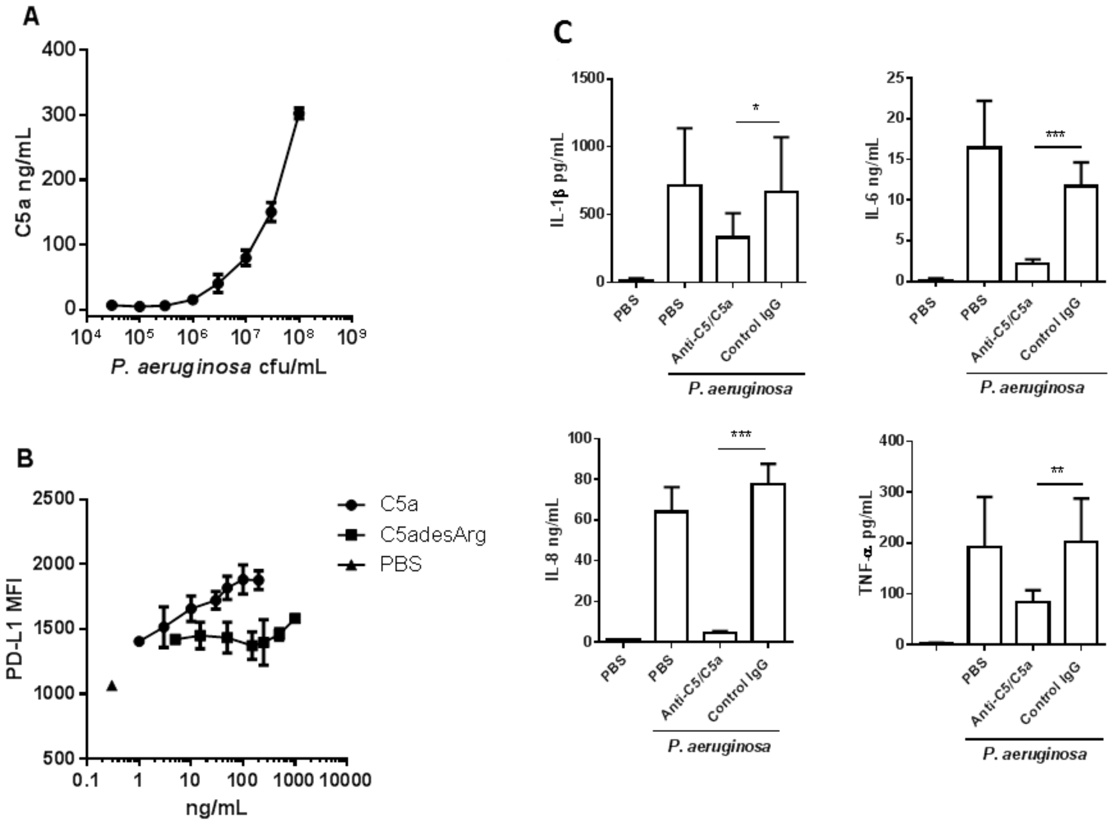

Supplementary Figure 2

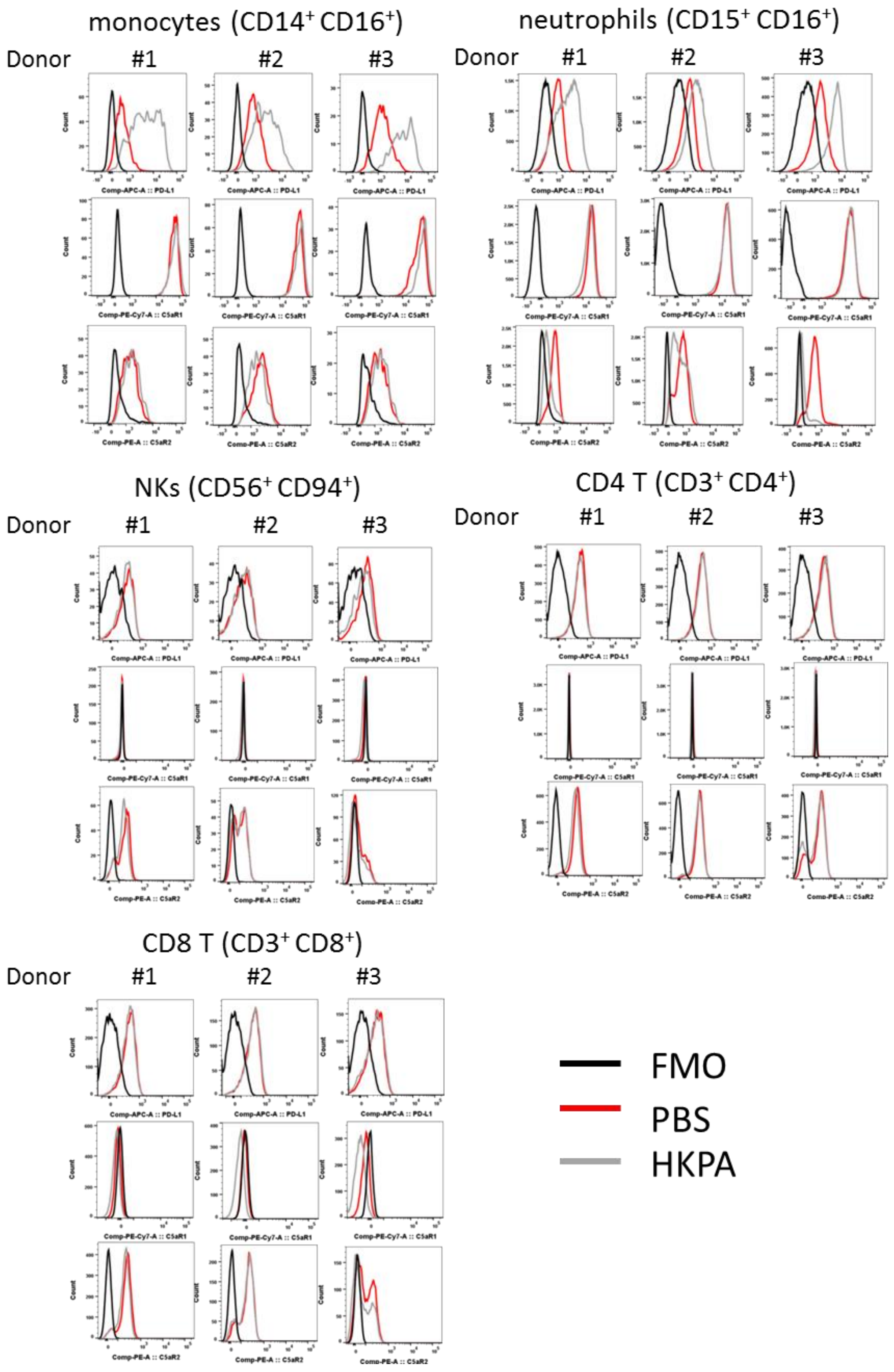

Table 1

**Cell type**	**PD-L1**	**C5aR1**	**C5aR2**
monocyte	-	-	↑↑
neutrophil	-	↓	↑↑
NK cell	-	-	-
CD4 T cell	-	-	-
CD8 T cell	-	-	-

Supplementary Figure 3

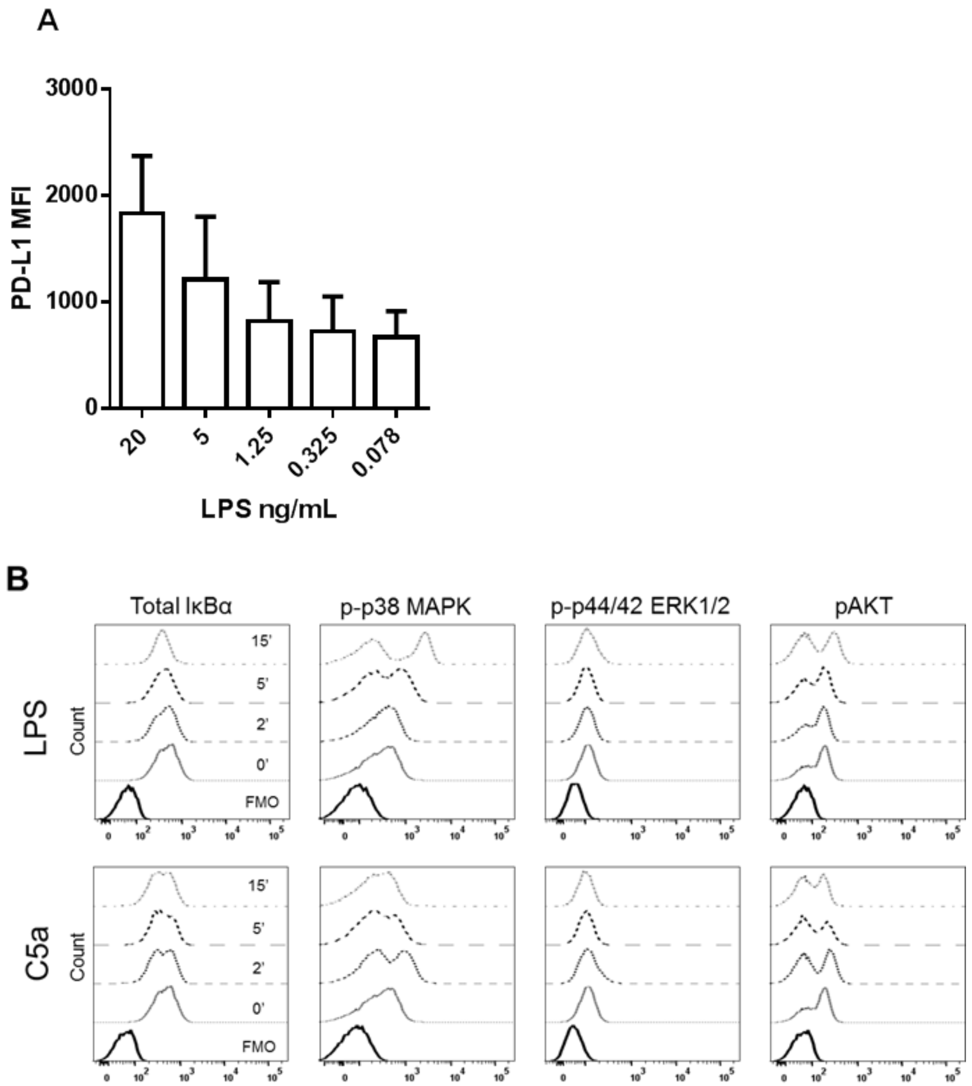

Supplement: Supplementary Information [file srep33346-s1.pdf]
